# Flow Rate Control by Means of Flow Meter and PLC Controller

**DOI:** 10.3390/s21186153

**Published:** 2021-09-14

**Authors:** Sylwia Włodarczak, Marek Ochowiak, Michał Doligalski, Bartosz Kwapisz, Andżelika Krupińska, Marcin Mrugalski, Magdalena Matuszak

**Affiliations:** 1 Department of Chemical Engineering and Equipment, Poznan University of Technology, 60-965 Poznan, Poland; sylwia.wlodarczak@put.poznan.pl (S.W.); marek.ochowiak@put.poznan.pl (M.O.); bartosz.kwapisz@student.put.poznan.pl (B.K.); andzelika.krupinska@put.poznan.pl (A.K.); magdalena.matuszak@put.poznan.pl (M.M.); 2 Institute of Metrology, Electronics and Computer Science, Faculty of Computer, Electrical and Control Engineering, University of Zielona Gora, ul. Licealna 9, 4, 65-417 Zielona Gora, Poland; 3 Institute of Control and Computation Engineering, Faculty of Computer, Electrical and Control Engineering, University of Zielona Gora, ul. Licealna 9, 4, 65-417 Zielona Gora, Poland; m.mrugalski@issi.uz.zgora.pl

**Keywords:** flow meter, PLC, algorithm, flow rate, measurement, control, logic controller

## Abstract

This paper presents a design of a flow meter based on a programmable logic controller (PLC). The new construction of a flow meter controlled by PLC increases the possibilities for the control and automation of fluid flow. Additionally, the didactic potential of the use of simple automation in the form of a programmable logic controller was considered. A device enabling the measurement of fluid flow rate based on a PLC controller was designed, constructed, and tested. The choice of device was the Gems Sensors FT-210 series turbine flow sensor, which is characterized by low purchase and maintenance costs. The properties and the chemical resistance of polyamide-12, the material the sensor is made of, make it possible to test the flow of various types of fluids. As part of the work, an algorithm and a program controlling the device was developed based on the APB Soft software, enabling the accurate reading of the number of impulses sent by the turbine flow sensor. The results of the designed flow meter were compared with the results obtained for the Krohne VA-40 high accuracy rotameter.

## 1. Introduction

Controlling fluid flow requires constant flow monitoring and the measurement of flow rates [1,2,3]. Problems that occur in flow meters can cause product quality and efficiency to decrease. For this reason, it is important to identify potential issues in order to prevent them from occurring and to take the appropriate measures to ensure proper flow measurement [4,5]. One of the more commonly used flow meters is the turbine flow meter [4]. It uses a multi-blade rotor to sense the average fluid flow rate. It has the advantages of high precision, good repeatability, a simple structure, high pressure resistance, a wide measuring range, small volume, and long durability [4]. Additionally, there is no need to scale the meter when measuring the volume of the flowing fluid, as is the case with rotameters [6,7]. The modern construction of flow meters, controlled by a PLC (programmable logic controller) with a high measurement accuracy, increase the possibilities of control and automation related to fluid flow [1,8], including the inclusion of single board computer software [9]. Process variables, such as temperature and flow rate, can be monitored via an input module that is typically part of the PLC [10]. Process data, diagnostics, commands, parameters, calibration data, and other information can be passed down to the sensors and actuators. The proper control of the liquid flow has a multidimensional meaning [11], especially, in particular, in terms of reducing the energy used [12].

At present, an important factor for the economy is the competitiveness of companies in their relevant industry branches. In order to stand out from the competitors, a company uses variable factors, including product quality, its price, and availability. One of the ways to influence these factors is to automate the production process by using solutions such as control systems and automatic regulation systems. Automation makes it possible to replace the direct involvement of human beings in the performance of simple, repetitive, and sometimes even tedious production activities, which often require high precision and repeatable results, and consequently allow them to be assigned to general control tasks [13,14]. The application of PLCs allows for the implementation of reliable control systems and their integration into distributed binary control systems [10]. The developed flow control algorithm, which can be implemented in PLCs, has special application significance [15,16]. In industrial production or control systems, PLCs play a key role. Standardization through the use of a PLC platform, followed by the implementation of a control algorithm, enables the integration of the flow control system and the further use of fault tolerant algorithms [17]. The implementation of binary control in a dedicated hardware platform in a broader perspective also enables the implementation of system diagnostics—both in the context of the flow control itself and in terms of the controller as part of a larger control system [18]. For the above reasons, when designing an apparatus, it is necessary to introduce the possibility of controlling and automating technological processes [19,20]. By definition, flow meters are devices used to measure the amount of fluid flowing through them or its flow rate [13,21]. Each flow meter is constructed in such a way as to enable the collection of readings over a specific fluid flow range. The upper limit of this range is the flow meter permeability, which is the maximum flow rate that the meter can test under normal operating conditions. The lower limit is the lowest accuracy value beyond which the error indicated by the flow meter is unacceptable [1,4,5]. Moreover, each flow meter has a characteristic size, which is the mounting diameter, i.e., the diameter for which the flow meter is intended [13,21]. There are various types of turbine flow meters, however, a basic principle that they all share is that the flowing fluid engages the rotor/impeller or any other mechanical system, causing it to rotate, and the rotation movement is then converted into a digital signal [21]. The main component of vortex flow meters is the rotor, which rotates due to the fluid flow. The calculation basis is the number of revolutions, which is proportional to the average flow velocity. It is defined as:

(1) $$n=c\cdot v_{av}$$

where *n* is the number of rotor revolutions; *c* is the proportionality coefficient, which accounts for the mechanical and hydrodynamic properties of the device; and *v_av_* is the average velocity of the liquid flow (m/s).

By inserting the formula for the volumetric flow rate into this equation, we obtain:

(2) $$n=c\frac{\dot{V}}{A}$$

where $\dot{V}$ is the volumetric flow rate (m^3^/s); and *A* is the conductor cross-section area (m^2^).

The discharge in any channel section can be computed by the equation:

(3) $$w=\frac{\dot{V}}{A}$$

where *w* is the mean velocity for the cross section (m/s). Cost-effective continuous measurements of mean channel velocity and cross-sectional area often cannot be made, and, thus, mean channel velocity and cross-sectional area must be estimated using calibrated relations with in situ velocity (index velocity) and stage measurements. In the analyzed case, the volumetric flow rate and the cross-sectional area of the channel are known.

From Equation (2), it can be concluded that the number of rotor revolutions is proportional to the volumetric flow rate of the liquid. As a result, we simultaneously obtain the measurement of the amount of liquid that has flown and its velocity [19,22]. The idea of a new construction for a flow meter controlled by PLC was based on the article [9]. Ali et al. [9] attempted to fabricate a turbine flow meter so as to obtain digital readings, along with temperature readings, using Arduino software. The effect was satisfactory. The subject of the present work is the design and construction of a complete flow meter that is controlled by a PLC, from the Taiwanese company Array Electronic Co., which will enable the accurate measurement of liquid flow rate [23]. This paper explores the use and benefits of embedding gas/liquid flow computer technology into PLCs for integrated measurement, control, and communications. Accurate measurement of gases/liquids using industry measurement standards is vital for process control systems, leak detection, and the transfer of product between two parties, which is more commonly known as custody transfer. As the complexity and scale of energy systems increase, automation technologies become more important for production efficiency and controlling costs. An online monitoring experimental platform using hardware-in-the-loop simulation, based on PLC hardware and the Kingview detection system, is presented in work [24]. Process control engineers now have new innovative automation technologies for flow measurement, control, and data management [9,23,24].

## 2. Materials and Methods

At the Department of Chemical Engineering and Apparatus at Poznan University of Technology, a PLC-based flow meter design was developed. Additionally, the didactic potential of the application of simple automation, in the form of a programmable logic controller, was taken into consideration. Last year’s PLC technology was significantly improved to support many functions well beyond discrete control and simple data collection. Modern PLCs include many process control functions (such as process identifier (PID) loops and recipes), while offering a broad range of communication options and data management tools. This has led many to now refer to PLCs as programmable automation controllers, or PACs. The key is that PLC processor power, which is near real-time I/O speed and flexible networking options, make the PLC platform ideal for just about any challenging industrial application, including accurate flow measurement. PLCs are electronic circuits, built on the basis of a microprocessor, that perform their functions in accordance with the program stored in their memory. They are divided into modular controllers (with the possibility of hardware reconstruction, for example, by adding modules such as inputs, outputs, central unit) and compact controllers (used only when they meet the process requirements). Due to the modular structure of these drivers, as well as the ease of changing the program being executed, they prove themselves in many different applications [23].

**Figure 1:**
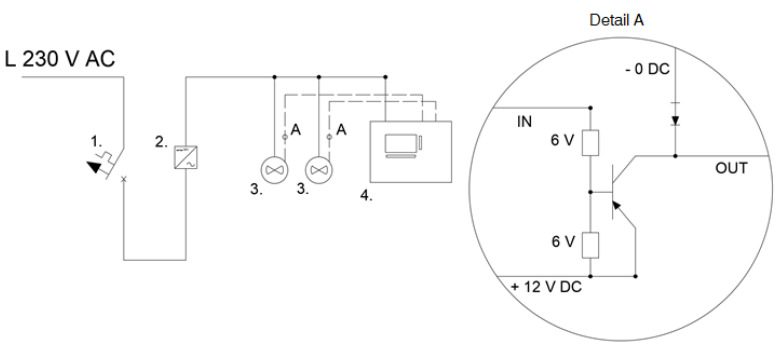
Electrical diagram of the system: (1) B6 overcurrent fuse, (2) ZI-20 power supply, (3) FT210 flow meters, and (4) PLC APB-12MRDL.

Figure 1 shows the electrical diagram of the meter-controller system. The circuit consists of two main parts: the actual meter and the controller that processes the signal sent. The polarity of the signal from the sensor is negative (shorted to the negative pole), while the PLC accepts digital signals with a voltage above 5 V but with a positive polarity (shorted to the positive pole). Hence, a PNP transistor should be used—the base receives a negative signal, conducts to the emitter, and this sends a positive signal. Before it was designed, requirements were established and, at the same time, the maximum budget for its preparation was set at EUR 200. The main feature of the system is characterized by its reliability. It was assumed that this meter would be used for both research and teaching purposes, and, therefore, it must withstand long periods of uninterrupted operation and repeated use (including incorrect use). In addition, the controller itself should be separated from the surroundings in order to protect it against accidental flooding and to protect the user against a possible short circuit and electrocution in the event of flooding. The system should also be easy to use, which will preclude the necessity for complicated training. The last criterion that had to be taken into consideration was the lowest possible purchase, preparation, and operation costs. Exemplary prices of the rotameters included the VA40V/R DN251 63–630 L/h for EUR 309; the VA40V/R DN501 630–6300 L/h for EUR 488, and the VA40V/R DN153 0.5–5 Nm^3^/h for EUR 269 – delivered by Krohne Polska Sp. z o.o. (Poland). The cost of a PLC is about EUR 50–100, while a sensor costs EUR 70. The total cost of the system (with one sensor of flow) was approximately EUR 150.

**Figure 2:**
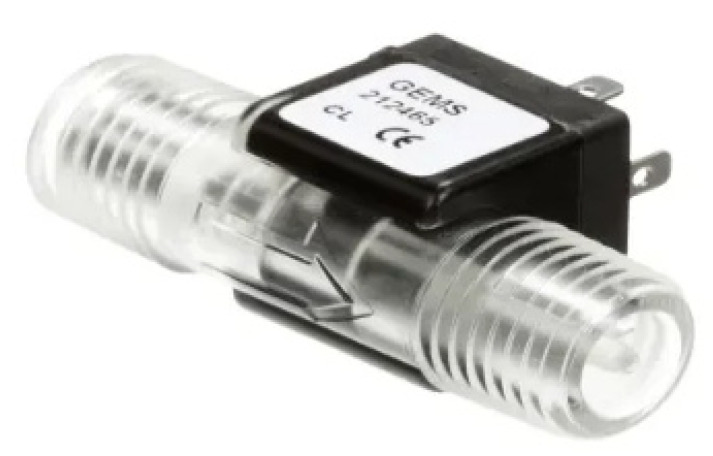
FT-210 series turbine flow sensor.

Each of these requirements was satisfactorily met. The selected PLC was the APB-12MRDL controller, which is relatively inexpensive compared to all other options of this type on the market, and, at the same time, ensures reliable operation and is an extremely good value for money. Its main advantage is a simple programming language that allows (after a quick grasp of the basic rules) for the creation of simple, useful applications for controlling any system. In the case of the meter, the Gems Sensors and Controls FT-210 series turbine flow sensor was selected (Figure 2). It is an inexpensive meter compared to the options presented in this paper, and, at the same time, it is characterized by high measurement accuracy, regardless of the physical properties of the tested fluid. As a result, the system can be used to measure a wide variety of flows in the laboratory without the need for pre-calibration. Water resistance and separation of the system from the environment was achieved via waterproof casing and the use of cables of appropriate length, which allowed the meter to be placed in a relatively safe zone away from risk.

**Figure 3:**
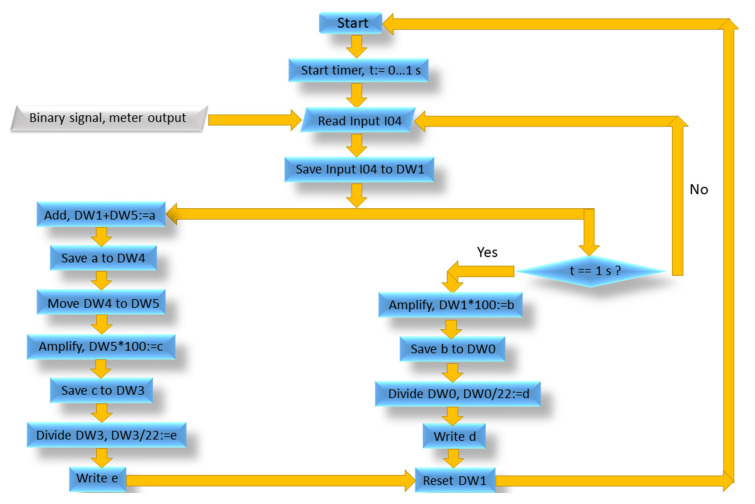
Algorithm for PLC firmware.

The algorithm for the operation of the PLC firmware, presented in Figure 3, was used to measure liquid flow. The algorithm consists of the following steps:

1. Start the algorithm;

2. Set timer in the range of 0 to 1 s;

3. Read the binary signal on the PLC’s Input I04;

4. Save the binary signal on the PLC’s Input I04 to the PLC’s internal registry DW1.

The next two paths are performed simultaneously:

Left path:

A loop is created in order to register all impulses sent from the meter since the start of the measurement, providing the whole flow volume. Start of the loop.

5.1. Add the value from the PLC’s internal registry DW1 to the value from the PLC’s internal registry DW5, thus creating value “a” in the RAM of the PLC;

5.2. Save value “a” to the PLC’s internal registry DW4;

5.3. Move the value from the PLC’s internal registry DW4 to the PLC’s internal registry DW5. End of the loop. To convert the impulses to flow reading, mathematic operations are required;

5.4. Amplify the value from the PLC’s internal registry DW5 by 100, creating value “c” in the RAM of the PLC;

5.5. Save value “c” to the PLC’s internal registry DW3;

5.6. Divide the value from the PLC’s internal registry DW3 by 22, creating value “e” in the RAM of the PLC;

5.7. Write the ”e” value on the PLC’s screen.

Right path:

These steps provide the value of current flow.

5.a. Check the timer, if t = 1 s, continue; if not, return to the Step 3.

To convert the impulses to flow reading, mathematic operations are required;

5.b. Amplify the value from the PLC’s internal registry DW1 by 100, creating value ”b” in the RAM of the PLC;

5.c. Save value ”b” to the PLC’s internal registry DW0;

5.d. Divide the value from the PLC’s internal registry DW0 by 22, creating value ”d” in the RAM of the PLC;

5.e. Write “e” value on the PLC’s screen;

6. Reset the PLC’s internal registry DW1 and return to Step 2.

**Figure 4:**
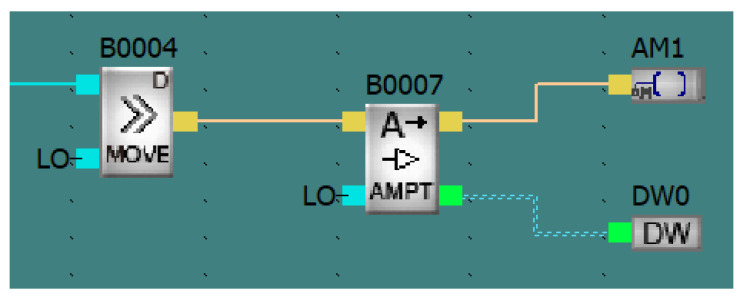
Stage 2 of the flow measurement—screenshot from the software.

The meter transmits a signal in the form of pulses, where each pulse stands for 1/22 ml of liquid. Next, the number of pulses is counted in intervals of one second, and, after appropriate calculations, the flow rate and the total volume of fluid that has flowed through the device are obtained. In order to read the measurement, the APB-12MRDL logic controller was applied, which was programmed with the use of function blocks in the APB Soft environment. The simplicity of the programming allows students to create uncomplicated programs. In order to accurately read the number of impulses sent by the vortex flow meter, a device control program based on the APB Soft software was developed. The device control program was written for the purposes of accurately reading the number of impulses sent by the vortex flow meter. Each impulse is a full revolution of the rotor, caused by the flow of exactly 1/22 ml of fluid as declared by the manufacturer in the catalog card. The time of collecting impulses is one second. In the first stage, the controller collects binary signal impulses from the meter connected to the input I04, which are then counted by the block B0001 and recorded in the DW1 register. Meanwhile, block B0002 is counting the time of collecting the measurement from input I04 every second and resets the state of the register DW1 through the internal input M0. The B0004 block transfers data from the DW1 register to the B0007 block (Figure 4), where they are amplified for the purposes of calculations. The result is transferred to the DW0 register. From the DW0 register, the result is taken by B0009 block, which is then converted to actual fluid flow in mL/s. The L0 block takes the result from the AM0 output and displays it in the top line of the controller display.

**Figure 5:**
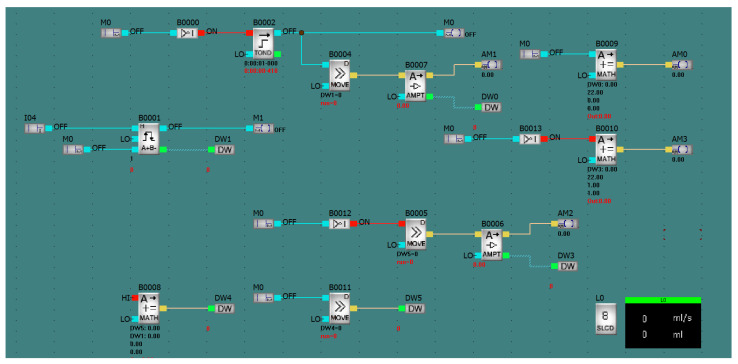
Embedded algorithm simulation.

The second function of the program is counting the volume of the fluid that has flown through the measuring system during the measurement, which allows for the determination of the specific fluid consumption in a given circuit of the installation. In stage 1, block B0008 adds together the DW1 register with DW5, which provides the number of impulses that have flown since the start of the measurement, and the result is recorded in the DW4 register. Block B0011, on the other hand, shifts the value from DW4 to the DW5 register. This is because when the cycle starts, the state of the calculations from before that cycle are recorded in the DW5 register. Due to this, the continuity of the measurement is maintained, which allows the PLC to save the entire state. In the next stage, B0005 block transfers the value from the DW5 register to block B0006 where it is amplified. The result is recorded in the DW3 register. Then, the B0010 block translates the value from the DW3 register to the appropriate measurement unit. In the last stage, the L0 block takes the result from the AM3 output and displays it in the bottom line of the controller display. Figure 5 shows the course of the entire program, together with the simulation of its operation.

**Figure 6:**
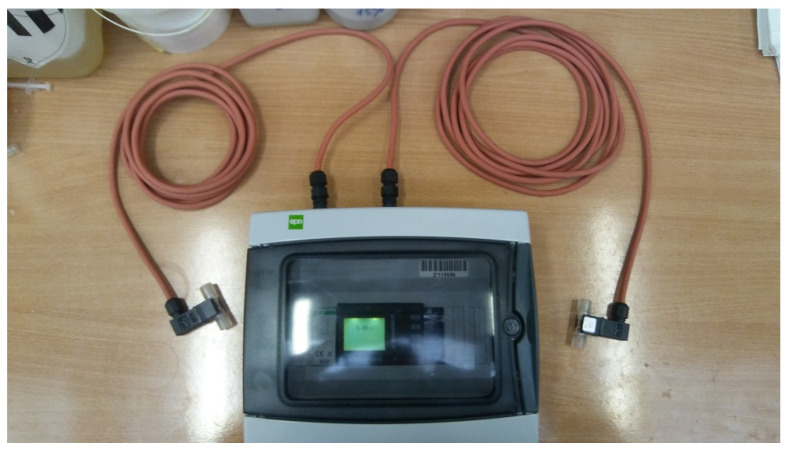
The PLC controller with flow meters.

Figure 6 presents the inside of the meter, where the controller wiring and components of the system are visible. On the left, an over-current fuse, which serves as a switch, the power supply, and the PLC are displayed. The assembly was facilitated by a steel mounting rail, intended for the arrangement of fuses, relays, and other components of the electrical network. The basic measuring system was equipped with two flow meters. The device allowed for the simultaneous connection of up to eight meters, each of which could be of a different type (for different measuring ranges of flow rates).

## 3. Results and Discussion

In order to validate the measurements, the results of the meter-controller system were compared with the results obtained from the Krohne VA-40 high accuracy rotameter battery and with the results of water volume measurements over time using a measuring cylinder (Table 1). The uncertainty of the VA-40 is ±1.0% (acc. to VDI/VDE 3513, Sh. 2). In conformity with VDI/VDE 3513, Sh. 2, the accuracy for variable area flow meters is defined by various accuracy classes. The following total errors are permitted as a factor of the flow rate, measured as the % of measured value or the % of full-scale range:

(4) $$F=\left(\frac{3}{4}M+\frac{1}{4}E\right)K\;\left(\%\right)$$

where *F* is the total error in flow units, *M* is the measured value in flow units, *E* is the full-scale value in flow units (Table 2), and *K* is the figure specified as the accuracy class (*K* = 1 for VA-40).

**Figure 7:**
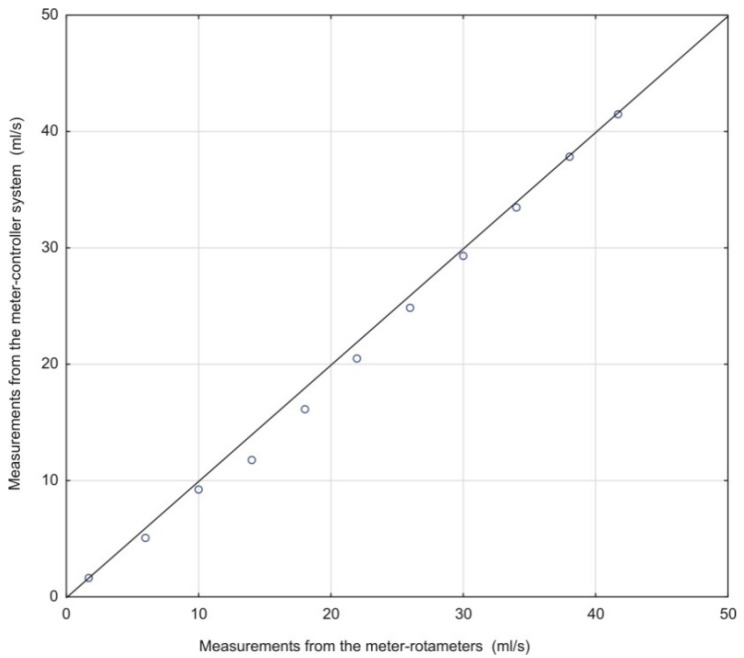
Correlation diagram of measurements from the meter-controller system vs. reading from rotameters.

Figure 7 shows a correlation diagram of the measurement results of the flow rate for the meter-controller system on the readings for rotameters with a marked linear regression. It was calculated that, with the assumed confidence level of 95%, for the constant *a* in the linear regression equation, the standard error was 0.02. The obtained value of the Pearson linear correlation coefficient *R* = 0.999 confirmed the presence of strong positive correlations between the reading of the fluid flow rate from the rotameters and the reading of the flow rate using the constructed meter.

**Figure 8:**
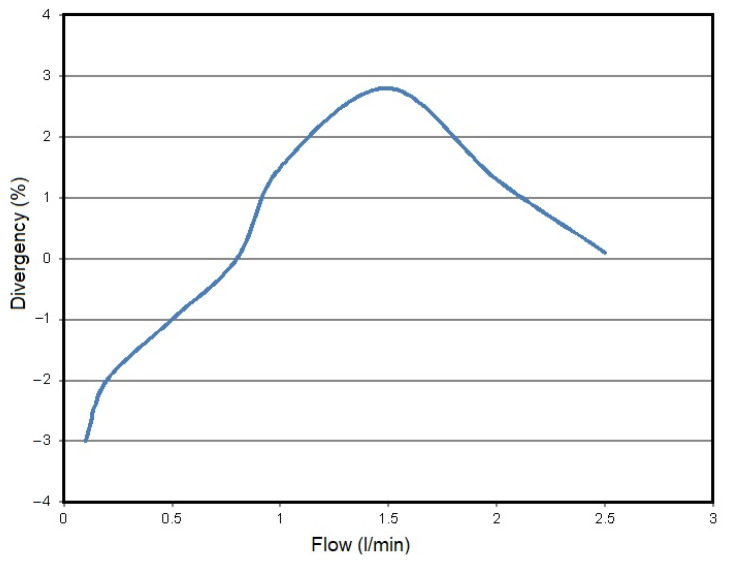
Correlation diagram of divergency vs. liquid volume flow rate.

For the flow of 1 L of liquid, the meter sends 22,000 pulses. This can be easily converted into interesting values For example, as the declared range of flows is known to be 0.1 to 2.5 L/min, the minimum frequency of pulses is 36.6 Hz, while the maximum is 916.66 Hz. In terms of measurement uncertainty, the accuracy is at the level of ±3% of the measurement, and the deviation is shown in Figure 8. As for the dependence on external conditions, temperature issues have a very negligible influence on possible signal transmission issues, as the minimally increasing resistance does not interfere with the transmission of signals. The only limitation is the operating range of the meter, i.e. from −20 to 100 °C. However, in this range all deviations are within the declared ones.

The producer recommends filtering the liquid stream before running it through the meter. Very small particles do not interfere with the measurement, but more concentrated colloidal solutions or large dirt particles may be a problem. The measuring system can, however, be washed as the meter itself is made of Grilamid TR55 and nylon, with the addition of ferrite.

The proposed solution is characterized by a direct reading of the liquid flow rate and, compared to rotameters, does not require calibration when changing the liquid. Additionally, there are no errors related to the relative reading of the position of a float as is seen with rotameters. The accuracy of the measurements is more than satisfactory, but it can be increased by using higher accuracy turbine flow meters in place of the FT210 flow meters.

## 4. Conclusions

In this work, a device enabling the measurement of fluid flow rate based on a PLC was designed, constructed, and tested. The developed algorithm and control program were implemented in an optimal way, which translated into the accuracy of the meter. There was no omission of impulses during the counting, thus there was no distortion of the indicated result. The entire system was designed to be ergonomic and the operation was simple, as intended. All the initial requirements for the designed device were met. The analysis of the results showed that the developed meter had a low measurement error, and the accuracy was high and comparable to other devices. The advantages of the measuring system include the:

− Simple construction;
− Simplicity of software changes;
− High-level accuracy of measurements;
− Wide operating range;
− Possibility of mounting practically anywhere.

Certain applications require measurement accuracy with respect to pressure and temperature independence, which cannot be achieved with rotameters. This is where the volume/mass flow meters come into their own. Alongside the advantages of the variable area flow meters, the devices offer extremely precise and rapid measurement with sensors. This system is a simple, inexpensive, and accurate solution that can, for example, support the process of educating students in the field of fluid mechanics, chemical engineering, and measurement automation.

## Figures and Tables

**Table 1:** Examples of measurement results, where $\dot{V}$ is the average flow rate in mL/s.

No. of Measure	$\dot{V}$ for PLC Meter	Deviation for Data Measured with PLC (%)	$\dot{V}$ for VA-40	Deviation for Data Measured with VA-40 (%)	$\dot{V}$ for Cylinder Measurements
1	9.27	−2.22	10	5.49	9.48
2	11.73	1.38	14	21.00	11.57
3	16.09	4.75	18	17.19	15.36
4	20.51	4.91	22	12.53	19.55
5	24.80	5.00	26	10.08	23.62
6	29.31	3.46	30	5.89	28.33
7	33.47	9.81	34	11.55	30.48
8	37.83	5.94	38	6.41	35.71
9	41.52	7.76	42	9.01	38.53

**Table 2:** Data for rotameters VA-40 (class 1).

Flow Rate (%)	*M*	*E*
100	1.000	1.000
90	1.028	0.925
80	1.063	0.850
70	1.107	0.775
60	1.167	0.700
50	1.250	0.625
40	1.375	0.550
30	1.583	0.475
20	2.000	0.400
10	3.250	0.325

## Data Availability

The data presented in this study are available on request from the corresponding author.
